# Collective Instance-Level Gene Normalization on the IGN Corpus

**DOI:** 10.1371/journal.pone.0079517

**Published:** 2013-11-25

**Authors:** Hong-Jie Dai, Johnny Chi-Yang Wu, Richard Tzong-Han Tsai

**Affiliations:** 1 Graduate Institute of BioMedical Informatics, College of Medical Science and Technology, Taipei Medical University, Taipei, Taiwan, R.O.C.; 2 Institute of Information Science, Academia Sinica, Taipei, Taiwan, R.O.C.; 3 Department of Computer Science and Information Engineering, National Central University, Taoyuan, Taiwan, R.O.C.; National Institute of Genomic Medicine, Mexico

## Abstract

A high proportion of life science researches are gene-oriented, in which scientists aim to investigate the roles that genes play in biological processes, and their involvement in biological mechanisms. As a result, gene names and their related information turn out to be one of the main objects of interest in biomedical literatures. While the capability of recognizing gene mentions has made significant progress, the results of recognition are still insufficient for direct use due to the ambiguity of gene names. Gene normalization (GN) goes beyond the recognition task by linking a gene mention to a database ID. Unlike most previous works, we approach GN on the instance-level and evaluate its overall performance on the recognition and normalization steps in abstracts and full texts. We release the first instance-level gene normalization (IGN) corpus in the BioC format, which includes annotations for the boundaries of all gene mentions and the corresponding IDs for human gene mentions. Species information, along with existing co-reference chains and full name/abbreviation pairs are also provided for each gene mention. Using the released corpus, we have designed a collective instance-level GN approach using not only the contextual information of each individual instance, but also the relations among instances and the inherent characteristics of full-text sections. Our experimental results show that our collective approach can achieve an F-score of 0.743. The proposed approach that exploits section characteristics in full-text articles can improve the F-scores of information lacking sections by up to 1.8%. In addition, using the proposed refinement process improved the F-score of gene mention recognition by 0.125 and that of GN by 0.03. Whereas current experimental results are limited to the human species, we seek to continue updating the annotations of the IGN corpus and observe how the proposed approach can be extended to other species.

## Background

Knowledge about the functions and behaviours of genes and proteins is the primary research interest of life scientists, which can assist in gaining advanced perception of the complex mechanisms behind biological phenomena. Take users of PubMed as an example. In addition to bibliographic queries (*e.g.* an author's name or article title), Dogan et al. [1] found that the most frequent PubMed searches were for genes and proteins. In contrast to a bibliographic query, a gene/protein query tends to return a large number of results due to the ambiguity of gene/protein names and the frequent use of abbreviations in such a query.

In an ideal information retrieval system, a user would simply input an entity name and receive search results clustered according to the different entities that share this name. One method to approach such a system is to include the results of entity recognition for the documents to be indexed. Although significant progress has been achieved in named entity recognition, however its results are still insufficient for direct use because of the wide array of synonyms and high ambiguity of name variations in names across documents [2]. For instance, a search for “tumor protein p53” in the Entrez Gene database returns over 400 proteins with the same name in over 20 species. When the same term is used to query GQuery, a global cross-database NCBI search engine, even more complex results are obtained, inferring that distinguishing the true identity of named entities is indeed an indispensable process. Recently, some advanced retrieval systems, such as BioContext [3] and EVEX [4], have begun to integrate entity normalization/disambiguation components e.g., GeneTUKit [5] and GenNorm [6] to deal with the ambiguity issues. Entity normalization goes beyond the recognition task by linking a textual entity mention to a knowledge base entry. Several preliminary results [7], [8] have demonstrated that such a disambiguation process can improve search quality. It can also help one manually curate a database [9] and index entries [10], facilitate links among data across resources [11], [12], and improve the online browsing experience [13].

**Figure 1:**
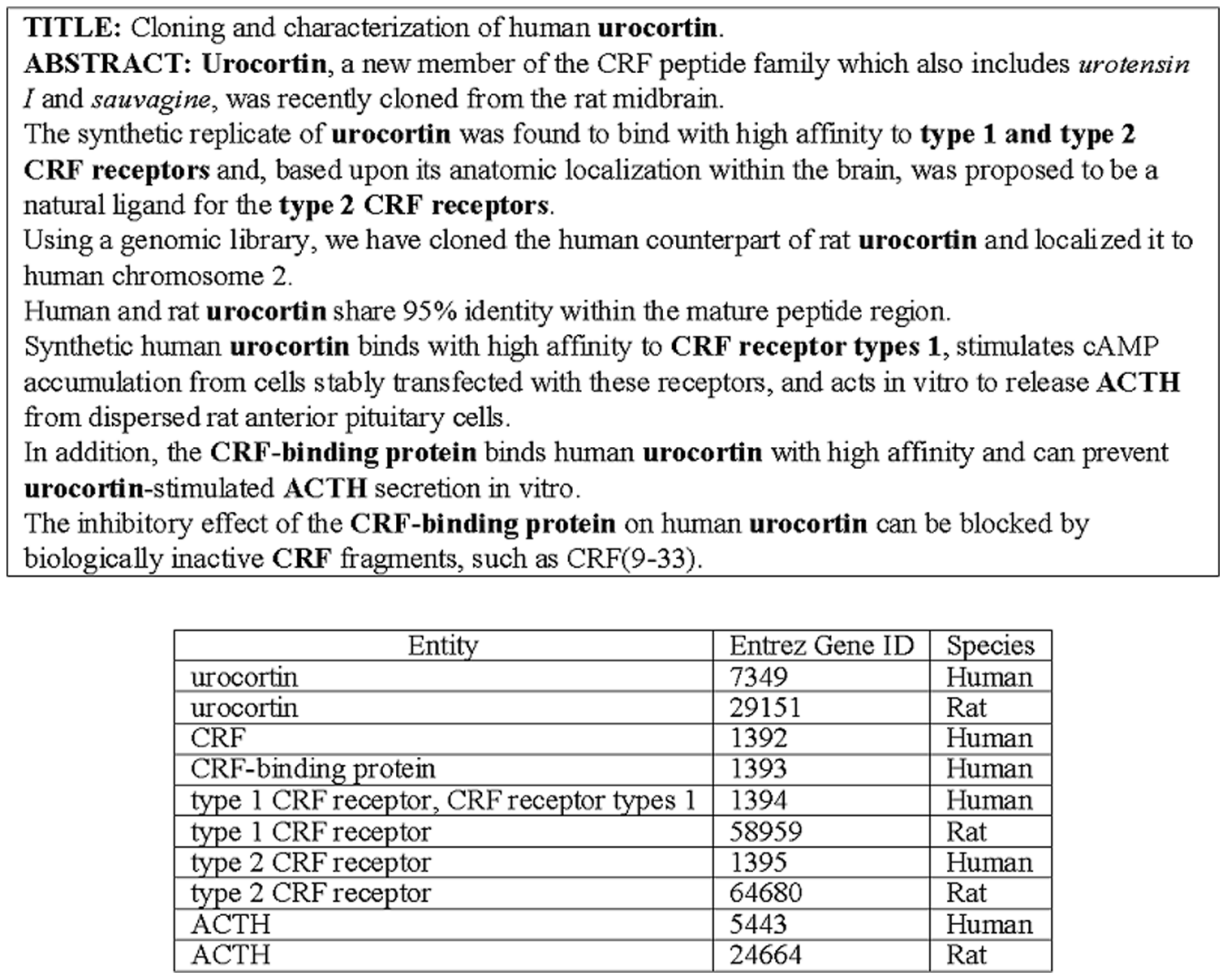
Examples of gene normalization (PMID: 8612563) and the result of the BioCreative Gene Normalization task . Note that for the explicitness of this example, the Entrez Gene IDs of rat genes are also included.

**Figure 2:**
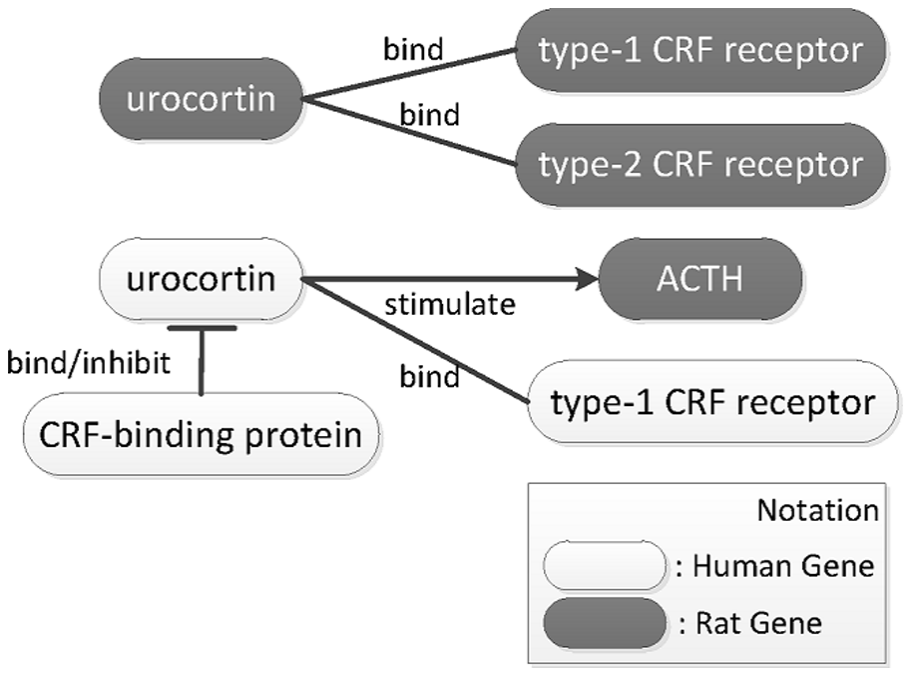
The pathway described in Figure 1.

This paper focuses on linking gene/gene product mentions in biomedical articles to their IDs recorded in a database, a task referred to as Gene Normalization (GN). Most previous GN-related tasks [14], [15] consider this task from the document-level perspective. For instance, in BioCreative II GN task [16], a normalization system is required to provide a list of gene mentions that exist in an abstract with their corresponding Entrez Gene IDs. Regarding the abstract shown in Figure 1 and its results after GN, the gene “urocortin” can be associated with multiple species in the abstract, as it appears in the abstract's title as a human gene, and as a rat gene in the first sentence of the abstract. Each instance must be linked to a different Entrez Gene ID. Unfortunately, the document-level linked result shown in Figure 1 is incapable of assembling the biological pathway shown in Figure 2, because it is difficult to distinguish which urocortins (7349, 29151 or both) are inhibited by the CRF-binding protein and/or stimulate ACTH. Only a few document-level GN systems, such as GenNorm [17], have begun to adjust their method by choosing the most consistent ID throughout the document-level set for bridging the extracted biomedical events and biomolecular database records. For the construction of biomedical pathways, linking biomedical entities in text to nodes in a pathway is a highly context dependent task [18] in which the precise identity of each of these entities should be recognized. Hence, an instance-level approach will serve as a better solution during the construction of biomedical pathways.

To this end, this work considers the GN task from the perspective of the instance level. In contrast to document-level methods, an instance-level GN system deals with the finer level of granularity that must link all gene mentions in a text, and also precisely give their exact occurrence information. The results are very important since they allow following applications to infer the interrelationships among those linked entities. However, it is also more challenging than traditional GN tasks.

When considering the task from the instance level, the first challenge is the lack of context information for disambiguating each individual instance. Conventional GN approaches [6], [19] have focused on making an independent decision for each gene mention. The essential idea was to extract the discriminative features (e.g., species, chromosome, etc.) from the profile knowledge of a specific gene (e.g., the gene's Entrez Gene page,) then link each gene mention in a document by comparing the contextual similarity with each of its candidate referent entries. For instance, in Figure 1, the candidate IDs of the “urocortin” in the first sentence of the abstract include {7349, 29151}. Judging from the context, this mention should be a rat protein, so it is linked to 29151. As these approaches only exploit local features around each name mention, they are referred to as individual GN approaches in this paper.

A drawback of individual approaches is that, when they decide the linking ID of a mention, they cannot make use of information about the linked IDs and features of other mentions in the same document. Therefore, the urocortin in the second sentence of the abstract cannot be linked because the context (*i.e.* the sentence containing the mention) does not provide adequate information. Our previous work [20] proposed to model the relations among gene mentions across sentences to deal with the challenge. For example, in Figure 1, the two urocortin genes described in the first two sentences can be correctly linked if we can infer that they form a co-referent. We refer this approach as an intra-section collective GN method because it considers both a mention's individual features and its relations with other mentions in the same section. In this paper, we extend the idea to consider not only intra-section collective information but also cross-section collective information and section-specific properties to improve GN performance. In addition, a refinement procedure is proposed to provide a sort of feedback mechanism from the GN step back to the gene mention recognition step to improve the overall performance.

The second challenge of the instance-level GN is the lack of an openly available gold standard corpus for developing instance-level entity normalization systems. The corpora of the previous BioCreative GN-related tasks, including BioCreative I-III [14], [15], [21], [22], only provide document-level annotations. On the other hand, the CALBC corpus [23] for the concept identification task is a silver corpus that was compiled by integrating different systems' output through a voting scheme. Therefore, they are not suited for developing or evaluating advanced applications to associate extracted relationships between entities with correct IDs. To the best of our knowledge, only three pioneering works aggressively attempted to list all mentions' identities and made their datasets openly available. The first is Cucerzan's dataset [24], which was compiled from two different sources: Wikipedia and MSBC news stories. The second dataset is released by Kulkarni *et* *al.* [25], which was sampled from online news. Both dataset are compiled for the newswire domain. There is only one similar corpus available within the biomedical domain, the CRAFT corpus [26], which contains 97 open-access biomedical journal articles annotated with nine biomedical ontologies. Nonetheless, the articles of the CRAFT corpus are manually annotated with sophisticated guidelines, which may at times increase the ambiguity of the dataset (*e.g.* the same name can be annotated with different ontologies due to its surrounding context). Therefore, in this work, we undertook to compile a more straightforward instance-level GN (IGN) corpus, which fully annotates gene/gene products and links them to the corresponding Entrez Gene IDs.

In this work, the proposed method was trained on the released IGN corpus and its performance was evaluated using instance-level precision/recall/F-measure on both abstracts and full texts. In addition, the performance of other instance-level GN systems, including GenNorm [27] and Moara [17], has also been reported. The current evaluation is only based on human genes, due to the leading demand of linking gene names to the human genome. Although our evaluation focuses on human genes only, the datasets used for evaluation also contain place-holders for non-human gene mentions. We used the Markov logic [28] to jointly model the candidate selection and disambiguation stages in the GN decision to filter out non-human gene candidates. These non-human genes were also taken into consideration during evaluation.

## Materials and Methods

### The Instance-level Gene Normalization Corpus

The IGN corpus was compiled using two datasets, one for abstract and the other for full text-level evaluations. For each article, in addition to the annotations of all described gene/gene product mentions, the following annotations are included in our corpus: 1) The corresponding Entrez Gene ID of each human gene mention, 2) Species information of each gene mention, 3) gene full name/abbreviation pairs, 4) co-reference of gene mentions, and 5) sentence boundaries. The annotated corpus, which can be downloaded from https://sites.google.com/site/hongjiedai/projects/the-ign-corpus, is released in the BioC XML format [29] as a publicly available resources for other text mining systems.

**Figure 3:**
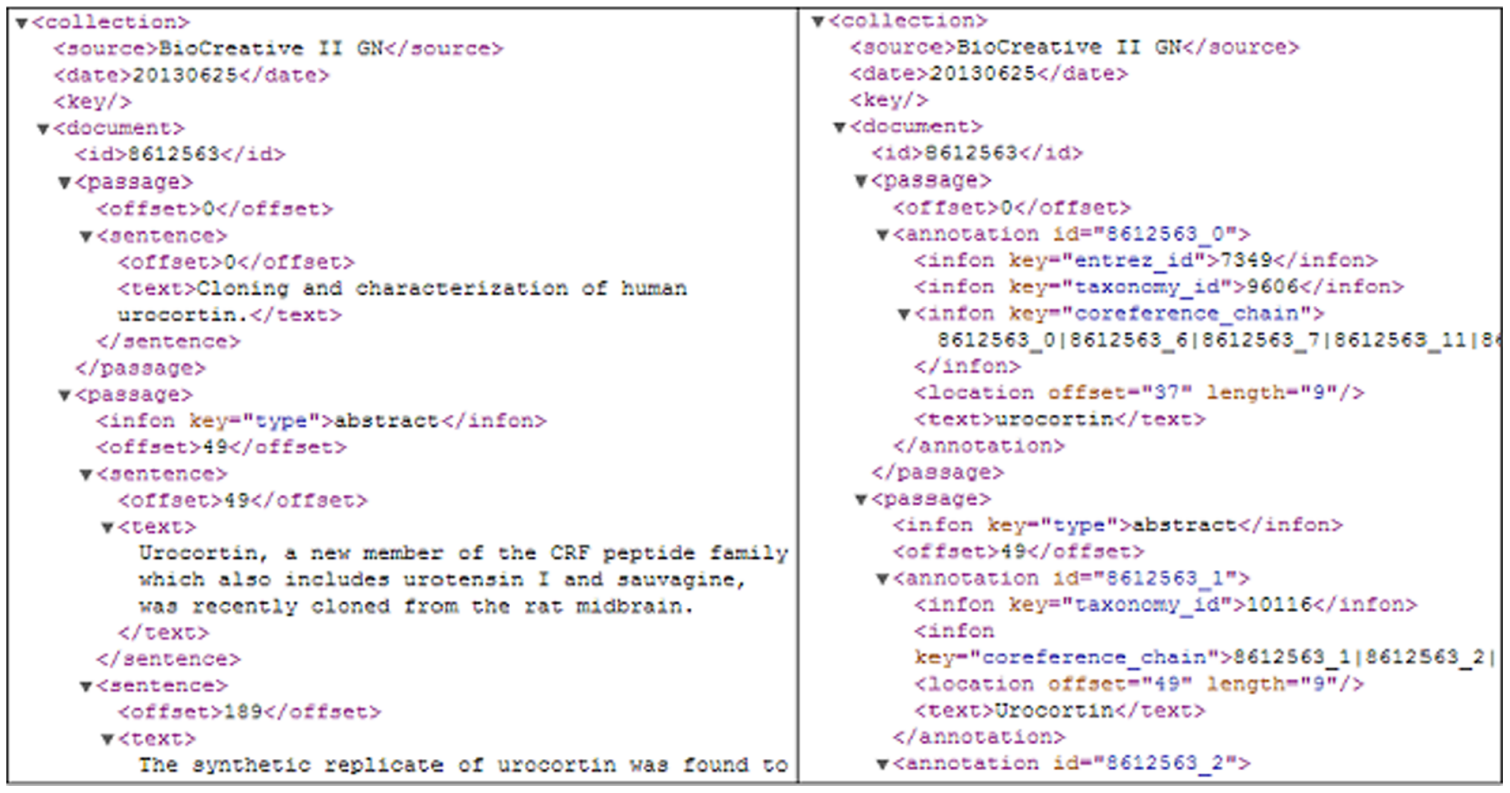
A snapshot of the IGN annotations in the BioC format.

Regarding the abstract level evaluation, we use the dataset released by the BioCreative II GN task [15]. For each abstract, the gold-standard BioCreative corpus contains a list of all human genes that appear in that abstract, and these name strings are linked to IDs in Entrez Gene. However, the lists do not give the exact location of the corresponding gene mentions in the abstract. To construct our IGN corpus, three in-lab annotators annotated the exact locations and the boundaries of the IDs' gene mentions by following an annotation guideline. Our annotations for the abstract shown in Figure 1 are displayed in Figure 3. All sentences within an abstract containing gene mentions are recorded individually, along with the precise boundary of these names. For those acknowledged as human genes, their corresponding Entrez gene IDs are specified (entrez_id). As for non-human genes, their probable associated species is provided (taxonomy_id). Furthermore, all instances within the article that possess the same identity are also listed out for reference (coreference_chain). Specific annotations as such are more suitable for the construction of signaling pathways than that of document-level annotations.

For the full text level IGN corpus, this work uses the dataset of the IntAct project, which was downloaded from ftp://ftp.ebi.ac.uk/pub/databases/intact/current/various/data-mining/. The IntAct dataset contains a set of 6,409 unique text snippets of about 1 to 3 sentences. Each snippet describes at least one protein-protein interaction that is encoded by using the IntAct interaction accession number. Through the PubMed IDs of the snippets, we retrieved all the corresponding abstracts and screened for matching snippets. If a snippet was found in its corresponding abstract, a pair of linked IDs of the interacting proteins in the given snippet was generated by resolving the accession number. For our evaluation, only human interaction pairs were selected, resulting in a corpus containing 45 abstracts with 59 snippets. Subsequently, the resolved IDs' name strings are used to tag the snippets, and then the annotators rectified the boundaries and ID linking errors based on the context. In addition, annotators were asked to complement the linking of gene mentions in the title section. After this process, sections containing snippets that are absent in the abstracts were manually located in the full text article and included into our corpus. 256 protein-protein interaction pairs were supplemented from 39 full-text articles.

### System and Methods

GN includes the two main tasks performed by a biologist reading an article: (1) gene mention recognition, and (2) gene mention disambiguation. The first task consists of recognizing words or phrases that are considered gene names. This task is a named entity recognition problem. The second task consists of finding the correct database ID that should be linked to a given candidate gene mention.

In this work, we employ several entity recognizers to locate biomedical entities, such as genes, tissues, chromosome locations and species terms, in a given text. After entity recognition, the gene mention mapping step assigns candidate Entrez Gene IDs to each recognized gene mention. A refinement step is then used to identify mentions that were not recognized by the recognition step. Finally, the gene mention disambiguation step selects the most likely ID from multiple IDs which share the same gene mention name. The following subsections elaborate each step in detail.

#### Entity Recognition

The recognition of gene names is handled by a machine learning-based gene mention recognizer trained on the BioCreative II gene mention dataset [21]. The gene mention recognition problem is formulated as a word-by-word sequence labelling task and the underlying machine learning model is the conditional random fields with a set of features selected by a sequential forward search algorithm [30]. In addition, a prefix tree string matching algorithm is implemented to recognize names of cells, tissues, and species mentions by using lexicons collected from online resources.

#### Gene Mention Mapping

In this step, we assign each recognized gene mention a specific Entrez Gene ID. This process requires a lexicon of gene mentions and related information such as name variations, acronyms, full names, spelling variations, *etc*. In this work, we use Entrez Gene and UniProtKB as sources to compile the lexicon. We use two mapping methods to assign each gene mention in an article with candidate Entrez Gene IDs. The first method is exact matching. Since the coverage of the dictionary is not sufficient to cover all gene mention variants, similar to the state-of-the-art GN systems, this work use rules to create orthographical variants for each name in the dictionary before matching. The second mapping method is partial matching. Gene names in the compiled dictionary are tokenized and indexed using Lucene. We then submit the recognized gene mention to our local Lucene index as a query term to find partial matches.

#### Gene Mention Recognition Refinement

**Figure 4:**
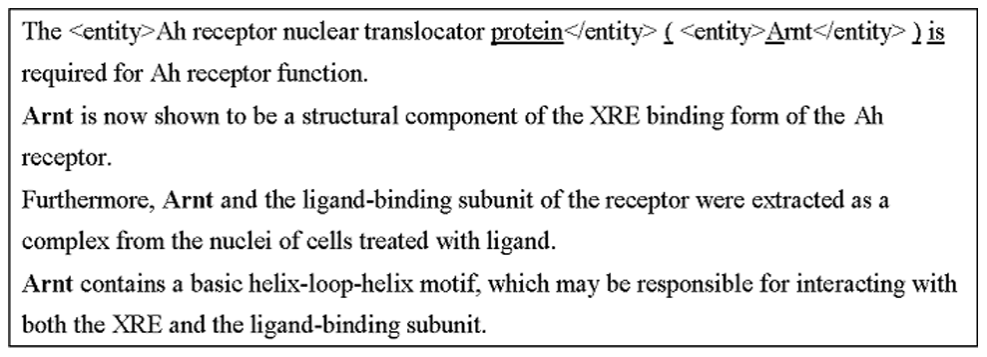
Problem of the supervised gene mention recognition. The tag <entity> indicates successfully recognized gene mentions. The context features extracted for the Arnt protein in the first sentence are underlined. The Arnt mentions that failed to be recognized are represented in the bold font.

After gene mention mapping, names of the successfully mapped gene mentions and their corresponding database IDs are collected to generate a refinement dictionary. Refinement is then performed by using the exact matching algorithm to search the whole article for mentions in this dictionary, which were not recognized by the recognizer. Figure 4 illustrates an example to emphasize the requirement of the refinement process.

In the first sentence of Figure 4, the protein “Arnt” was successfully recognized. However, in the following three sentences, the same surface name failed to be recognized by the employed supervised learning gene mention recognizer. As described in the previous section, the sequential forward search algorithm was employed to select the most effective feature sets. In the feature selection procedure, we found the following three features to be most effective:

1. Orthographical features: For example, “Arnt” matches the Initial_Captial pattern “∧[A-Z].+”.
2. Context features: because of the limited memory resources, the context window size of most entity recognizers was set to five, *i.e.*, the two preceding words (*i.e.* the words “protein” and “(” for the first “Arnt” mention), the current word (“Arnt”), and the two following words–“)” and “is”. The context provides the hint that “Arnt” is the abbreviation of a protein name.
3. Part-of-speech features: the context window was also set to five. For example, the part-of-speech of the protein “Arnt” is NNP.

These features provide sufficient information to imply that the surface name “Arnt” in the first sentence should be a gene product. However, in the other sentences, “Arnt” appears at the beginning of each sentence. Therefore, the effect of the orthographical features is reduced. Furthermore, the context does not provide any clues to support the conclusion that the word “Arnt” should be a protein. These issues make these mentions failed to be recognized. Our proposed refinement process in this step can avoid these types of errors.

#### Collective Gene Mention Disambiguation

If a gene mention is mapped to two or more database IDs, the disambiguation process is used to determine which one is more appropriate. We employ the collective classification method in this work. Collective classification refers to the task of inferring labels for a set of objects using not just their attributes but also the relations among them [31].

**Definition 1** Given a network *N*, an node *n* in *N*, and the label set *L*, there are three distinct feature types that can be utilized to determine the label *l* of *n*, where 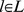:

1. The observed features of *n*.
2. The observed features (including observed labels if they are known) of nodes in the neighbourhood (related nodes) of *n*.
3. The unobserved labels of nodes in the neighbourhood (related nodes) of *n*.

A model that can classify a set of interlinked nodes or objects using all three types of information described above is referred to as a collective classification model. The main difference between our collective GN and individual GN is the model of the third feature; the dependencies among entities across sentences.

#### Formulation of the Collective Gene Normalization Problem

**Figure 5:**
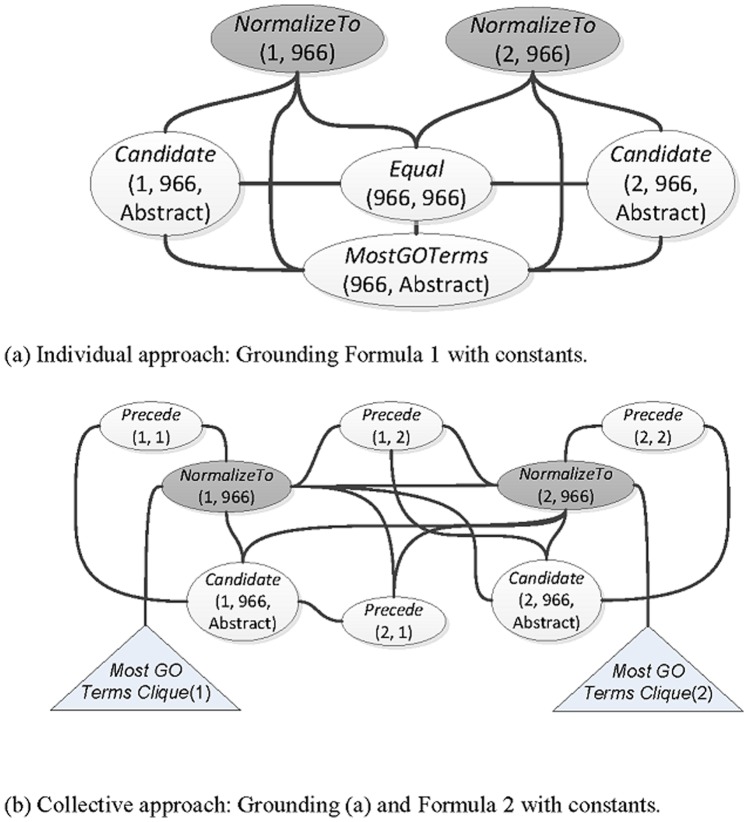
The Markov network obtained by grounding the discourse salience collective formula to the constants *x*, *y*  =  {1, 2}, *s*  =  {“Abstract”}, and *id*  =  {966}. Note that the circular nodes in dark background color represent the unobserved predicates, and the other circular nodes represent the observed predicates. The triangular nodes represent the cliques shown in (a).

In contrast to the individual GN formulations, which classify each mention's candidate IDs independently using features that describe the similarity between the current context and the database description of the given candidate ID, we formulate the candidate IDs of all recognized gene mentions in a given article as a network *N*. A node in *N* is constructed by using the properties of the candidate ID. For example, in Figure 5, the mention's ID (966) and its order (being the 1^st^ mention) form the node *NormalizeTo*(1, 966) in the network depicted in Figure 5 (a) and (b). An edge exists between two nodes if they have dependencies. For instance, it can be observed in Figure 5 that there are edges between the nodes *NormalizedTo*, *Candidate*, *Equal* and *MostGOTerms*, which implies that they are mutually dependent. In our implementation, the dependencies include the linguistic phenomena, discourse salience and transitivity; and the correlation among IDs, protein-protein interaction. The formulation is then implemented as a Markov network using the Markov logic [28], which alleviates the hard constraints of first-order logic by associating each first-order logic formula with a weight that reflects the strength of a constraint through the construction of a Markov network.

In our setting, we define the predicate *Candidate*(*x*, *id*, *s*) to represent that the gene *x* mentioned in the context *s* has a candidate Entrez Gene ID *id*; and the predicate *NormalizeTo*(*x*, *id*) to represent that *x* is linked to *id*. The priori which predicates will be observed (e.g. *Candidate* and *MostGOTerms*(*id*, *s*), which is true if the *id* has the largest number of corresponding gene ontology terms found in the context *s*) and which ones (e.g. *NormalizeTo*) will be queried is known, and the goal of the formulation is to correctly predict the hidden predicates (i.e. *NormalizeTo*) given the observed predicates. For instance, based on the first-order logic syntax and the assumption that a gene mention *x* should be linked to the *id* with the largest number of corresponding gene ontology terms found in the context *s*, we can define the following formula for individual-based GN where the symbol “
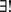
” indicates a uniqueness quantification. 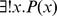 expresses that there is exactly one *x* such that *P*(*x*) is true.

**Formula 1:** 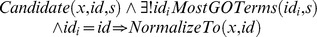


The constructed network of Formula 1 is shown in Figure 5, where no existing dependency is found between the two hidden predicates *NormalizeTo*. Therefore, we refer to formulae such as Formula 1 as individual formulae, because they didn't exploit the third information defined in Definition 1. Please refer to Material S1 for the list of employed individual formulae.

#### Intra-section Collectives

In our collective formulation, the linguistic phenomena and the correlation among entities are leveraged to build dependencies. The first phenomenon is the *salience collective*, which aims to capture the entity that is the center of attention in a given discourse. Such an entity is mentioned repeatedly, making it more salient than others. The collective can be expressed in the following formula:

**Formula 2: Salience Collective.** 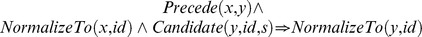


In other words, if the Entrez Gene ID *id* is linked to the *x*th gene mention that precedes the *y*th mention in the same section *s*, and *id* is a candidate ID of *y*, then the mention *y* should also be linked to *id*. In contrast with the individual formulae defined in Formula 1, which only considers the observed features of the target gene mention itself (*x*), Formula 2 includes features of neighboring gene mentions and their unobserved IDs. Figure 5 compares the constructed Markov networks of our collective GN approach and the individual approach. As shown in Figure 5, there are additional dependencies between the hidden predicates *NormalizeTo*. Based on the network, our collective entity disambiguation model can capture the dependencies among the unobserved IDs of gene mentions in the same context, allowing the information to be employed in the GN decision.

The second dependency is based on *transitivity collective*, which allows us to express the conditional claim that if two mentions *x* and *y* refer to the same concept, and one of the mentions is linked to an ID, then the other should also be linked to the same ID. The hidden predicate *Coreference*(*x*, *y*) is defined to capture the aforementioned conditional. We can then define the formula:

**Formula 3: Transitivity Collective.** 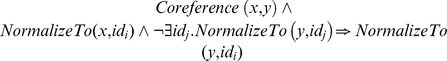


The transitivity collective formula states that if the *x*th and the *y*th gene mentions are a co-reference pair mentioned in the same context *s*, and *x* is linked to *id_i_* and *y* has not been linked, then *y* should also be linked to *id_i_*. In this work, we transformed a subset of features presented by Soon et al. [32] into Markov logic formulae to infer the co-references, and used the abstract level training set of our IGN corpus as the training set. Please refer to Material S1 to the full list of the implemented formula for the resolutions of co-referred pairs and human gene.

Finally, the protein-protein interaction collective is defined in the following formula, which states that the *y*th gene mention should be linked to *id_j_* if another gene mention *x* has been linked to *id_i_* and *id_i_* has an interaction with *id_j_* in the same context *s*:

**Formula 4: Protein-protein Interaction Collective.** 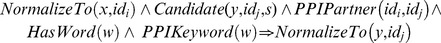


Lastly, since this work only concerns human genes, we need to integrate a human gene classifier into our collective model to ignore non-human gene mentions. To capture the concept in our model, we define the hidden predicate *HumanGene*(*x*), which indicates that the *x*th gene mention of the article is a human gene^5^. We then employ the following formula to ensure that whenever *x* is linked to an ID *id,* it must belong to the human species.

**Formula 5:** 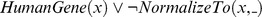 The symbol “_” in the predicate *NormalizeTo* (*x*, _,) indicates that the variable (*i.e. id*) can be any value. The rationale of the formula above is that the recognized gene mention *x* does not have to be linked to an ID. Nevertheless, the *id* cannot be assigned to *x* that has not been proposed as a potential human gene mention. The formula is defined as a hard constraint that must always hold. The species annotations of the IGN corpus is used to trained the human gene classifier.

#### Cross-section Collectives

In contrast to biomedical abstracts that summarize the content of articles, the full texts of papers contain more information of varying relevancy, which is much harder for a system to understand if it processes each section independently. For example, extracting facts from the Results section may require resolving acronyms or synonyms only mentioned in the Introduction section [33]. We propose advanced cross-section collectives to model the specific characteristics and structure of difference sections to improve the performance of GN. In brief, the idea is that GN results from information-enriched sections can improve results from information-depleted sections. For our purposes, sections with the most abundant information are those that are most likely to mention a gene's full name and contain the most background information about it. The introduction and abstract sections are usually the richest sections where the authors first mention the genes of interest, giving their full names–often followed by abbreviations used thereafter. In comparison, other sections and figure/table captions tend to comprise less information.

The basic collective GN formulae described in the previous section can be extended to use gene mentions linked in sections with sufficient information to help GN in later sections. For example, we add the predicate *InfoRichSection* in Formula 2, which is true for the section *s_i_* if *s_i_* is the abstract or introduction. The advanced salience collective is defined as:

**Formula 6: Advanced Salience Collective.** 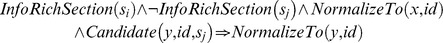


The basic salience collective links the ambiguous mention *y* to an *id* which has already been linked by another mention *x* that precedes *y* “in the same section *s*”. The advanced salience collective allows information about linked abstract/introduction mentions to be propagated across sections.

Following the same idea, the collectives defined in Formula 3 and 4 can be extended by including the *InfoRichSection* predicate as follows.

**Formula 7: Advanced Transitivity Collective.** 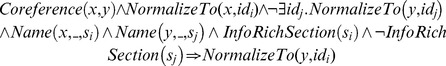


**Formula 8: Advanced Protein-Protein Interaction Collective.** 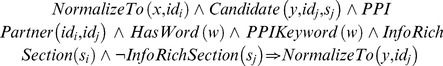


## Results and Discussion

### Evaluation Metrics

This work evaluates the performance of the proposed collective GN method by using the standard precision, recall, and F-measure metrics (PRF) at the instance level. The instance-level evaluation measures GN performance at a fine-grained resolution; the PRF scores are calculated based on the sums of true/false positive/negative counts of linked IDs for all gene mention instances.

### Experiments

We use the articles of our IGN corpus, which overlaps the training set in the BioCreative II GN task as the training set. The remaining articles are used as the testing sets in evaluating GN performance at the abstract and full-text levels. At both levels, we compare the assigned IDs of each human-annotated gene mention with the IDs determined by the GN system to calculate the PRF scores.

#### Experiment 1: The Effect of Refinement stage

Primarily, we examine the advance of the refinement stage to improve gene mention recognition and normalization performance. We use the abstract level IGN test set ignoring the linked IDs for each gene mention and apply the approximate boundary matching criterion to evaluate the recognition performance. We then add the refinement stage after gene mention mapping and reevaluate its performance on the same dataset. As shown in Table 1, adding the refinement stage can significantly improve the recognition PRF-scores. According to our analysis of the IGN corpus, for the results generated by the employed supervised learning recognizer [34], on average 19.5% of true gene mentions with the same name were not recognized by the recognizer in an article. Of these missed mentions, 29.7% appear at the beginning of a sentence or after a punctuation mark, and 12.1% of missed mentions only consisted of lowercase letters. The proposed refinement process reduces these types of errors. The result also presents the upper bound performance (77.6% in terms of F-score) following the GN stage.

**Table 1:** The effect of the refinement stage in human gene mention recognition on the test set of IGN.

Configuration	P (%)	Diff	R (%)	Diff	F (%)	Diff
Original	66.2	−	82.7	−	65.1	−
+Refinement	67.3	+1.1	91.8	+9.1	77.6	+12.5

#### Experiment 2: Basic Collective GN Performance

The second experiment compares the performance of the proposed basic intra-section collective methods with the individual approach and three baselines. We first conducted ten-fold cross validation on the training set of the abstract level IGN corpus to evaluate the performance of the proposed basic collective formulae (Formulae 2–4). The entire training set was then used to train a Markov logic network model for collective GN. Finally, its performance was evaluated on the abstract level test set.

Table 2 shows the experimental results. The first two rows are the performance of the two baseline systems without any disambiguation. For both, all mentions with only one candidate ID were directly treated as answers, and entities with more than one candidate ID were discarded (to optimize P; P-oriented) or kept (for maximal R; R-oriented). For each ambiguous gene mention, the third baseline “random baseline” randomly selects one candidate IDs as the linked ID.

**Table 2:** The performance of the intra-section collectives.

Configuration	Training set (%)			Test set (%)		
	P	R	F	P	R	F
No disambiguation (P-oriented)	80.4	48.6	60.6	80.7	56.3	66.3
No disambiguation (R-oriented)	64.7	56.3	60.2	51.2	73.3	57.7
Random baseline	68.4	51.6	58.8	68.3	59.8	63.8
Salience	79.2	50.2	61.5	79.5	59.0	67.7
Transitivity	78.5	49.5	60.7	78.6	58.8	67.2
Protein-protein Interaction	79.4	51.1	62.2	80.1	59.8	68.5
All intra-section collectives	79.1	52.0	62.8	78.4	61.0	68.6
All individuals	74.9	54.3	62.9	75.7	61.7	68.0

As shown in Table 2, all disambiguation configurations outperformed the random baseline by at least 3.4% (F-score) on the test set. The highest R-score was “R-oriented”, which applies no disambiguation processes and outputs all IDs as its linked results. All configurations that employed disambiguation rules improve the overall F-score at the cost of reductions in R. For instance, adding the salience collective without any domain knowledge can improve P by 28.3% and F by 10.0%. Protein-protein interaction collective with domain knowledge achieves the highest F-scores among the three proposed basic collectives. Table 2 also shows that adding all of the basic collective formulae can achieve an even better F-score than all individual formulae on the test set.

We further examine the effect of adding the refinement stage in GN by combining it with three GN methods: all individuals, all intra-section collectives, and all individual plus intra-section collective formulae on the test set. As shown in Table 3, the refinement process can significantly boost the R rate of GN and results in an improved F-score no matter what GN method is used. The results are reasonable, because the refinement process can significantly improve the R rate of the gene mention recognition process. Table 3 also shows the results of other instance-level GN systems, including GenNorm and Moara. Note that the results are listed here just to illustrate the state-of-the-art instance-level GN systems' performance on the IGN corpus. There is no intention of directly comparing these results, due to the fact that each of them is based on different gene mention recognition/mapping systems, respectively.

**Table 3:** The GN results after employing refinement process and the performance of the other two openly available instance-level GN systems.

Configuration	P	Diff	R	Diff	F	Diff
All Individuals^+^	72.0	−3.7	70.4	+8.7	71.2	+3.2
All Intra-Section Collectives^+^	77.4	−1.0	68.9	+7.9	72.9	+4.3
Individuals+Collectives^+^	77.8	−0.1	70.7	+5.4	74.1	+3.1
Moara (Exact Matching)	60.6	n/a	50.3	n/a	55.0	n/a
GenNorm	75.5	n/a	55.3	n/a	63.9	n/a

#### Experiment 3: The Effect of Cross-section Collectives

In this experiment, we evaluate the effect of the proposed advanced cross-section collective formulae on the IGN corpus. Because the abstract level test set in IGN only contains abstracts, we divided each article into abstract and title sections to simulate the informationally rich and poor sections, respectively. For the full text-level dataset, we treated sections other than the abstract section as informationally poor. This experiment then evaluated the performance of the two models trained on the training set: the basic collective GN model with all of the individual and intra-section collective formulae, and the same configuration plus the advanced cross-section collective formulae.

Currently, the full text-level dataset was not exhaustively annotated by our annotators. Therefiore, this work only evaluated the PRF scores for the annotated instances. Recognized mentions whose boundaries do not overlap with manually annotated ones are ignored in the evaluation. The upper half of Table 4 shows the results on the IGN corpus for full abstracts and titles, respectively. In full abstracts, we can see that adding the advanced collectives improves both P- and R-scores and results in a 0.3% improvement in F-score. The improvement is even greater for the informationally poor title section. Adding advanced collectives here boosts the R rate by 3.6% and improves the overall F-score by 1.8%.

**Table 4:** The effect of the advanced collectives on abstract and full text-level IGN.

Dataset	Configuration	Full Abstracts			Titles		
		P	R	F	P	R	F
Abstract-level IGN	Intra-section Collectives	77.8	70.6	74.0	82.3	62.5	71.1
	+Cross-section Collectives	78.0	70.9	74.3	81.3	66.1	72.9
Full text-level IGN	Intra-section Collectives	85.8	59.4	70.2	81.6	49.1	61.3
	+Cross-section Collectives	86.0	60.3	70.9	82.0	50.2	62.3

**Figure 6:**
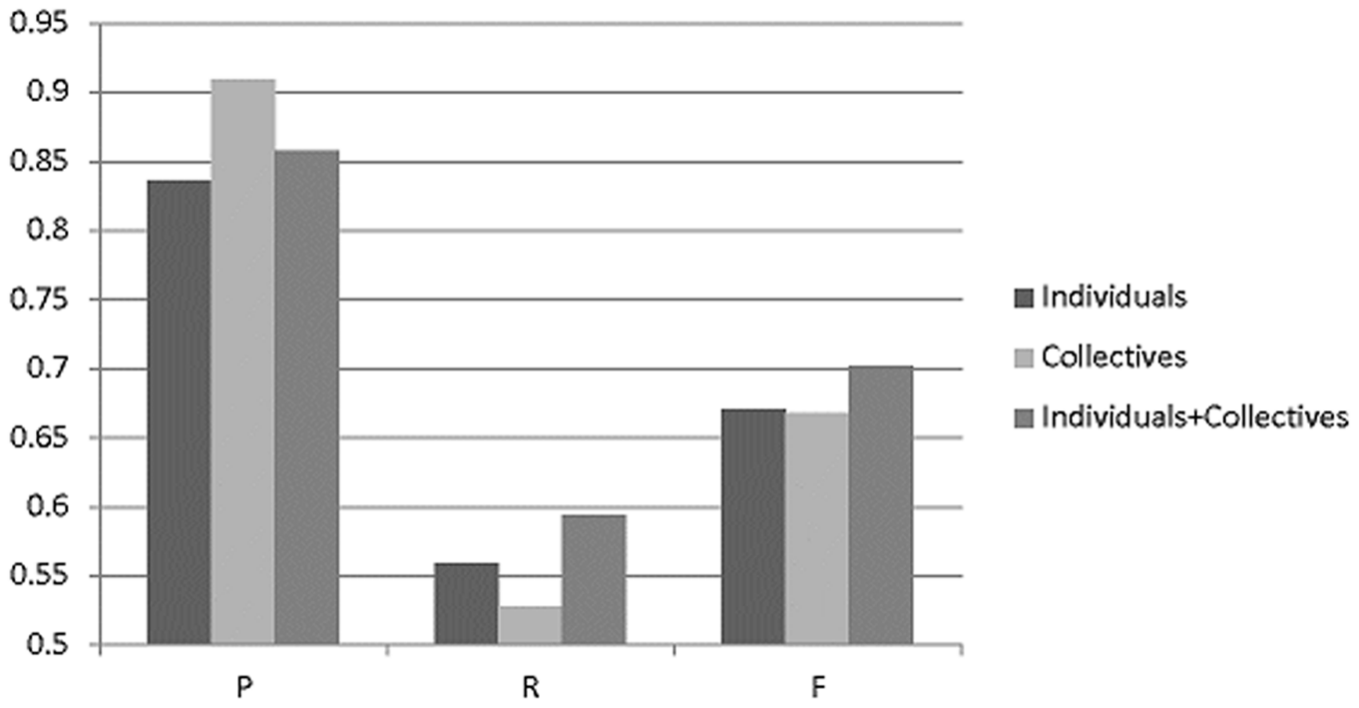
The performance of the individual and the collective methods on full text.

For the full text-level dataset, Figure 6 shows the performance of the three GN models: individual, collective and combined formulae. The results show that the performance of these models on the full text dataset is consistent with their performance on the abstract. The pure-collective model achieves a higher P-rate, the pure-individual model has a better R-rate, and the combined individual+collective model achieves the highest F-score. At the lower half of Table 4, we can see the improvement of the proposed advanced collectives over the basic collectives. Overall, Table 4 substantiates the effectiveness of the proposed advanced collectives.

#### Experiment 4: The Effect of Different GN Methods on Protein-protein Interaction Pair Extraction

As described in the Introduction, we believe that document level GN is insufficient for identifying the relations in an article. This experiment uses our IGN full text-level corpus, which is based on the IntAct dataset, to compare the linked protein interaction pairs of the document-level approach with the results of our instance-level approach. In this experiment, only protein interaction pairs that were explicitly described in one sentence are selected for evaluation. In total, 377 pairs are evaluated.

Three configurations are compared in the evaluation. The first and the second ones are based on our collective approach. In the first configuration, we directly take the linked results generated by our instance level collective GN approach. The second configuration simulates the document-level GN results by combining the results of the instance-based approach as follows: The system first collects the names and machine-linked IDs of all recognized protein interaction pair members. It then aggregates the machine-linked IDs for each name in the collection. Therefore, one name could be linked to more one ID. Finally, it assigns the aggregated IDs to all of those name mentions as their linked IDs. Therefore, the R rate of the second configuration should be better than the first. In the third configuration, our previous document-level maximum entropy-based GN approach [20] is employed.

Table 5 shows the performance when different GN methods were employed. The results show that the proposed instance-level GN approach achieves the best P-score on the event extraction task. This may be because the approach can provide the ID for each interactor. In contrast, the document-level approaches generally achieved better R rates. The results show the benefit of the instance-level GN method: the results of the instance-level GN can be easily transformed into document-level results (see the results of configuration (2)) and still achieve better performance (*cf.* the configuration (2) and (3) in Table 5).

**Table 5:** The effect of different GN methods combined with protein-protein interaction extraction.

Configuration	P	R	F
(1) Instance-level Collective GN	81.6	48.3	60.7
(2) Document-level Results of (1)	34.1	79.6	47.7
(3) Traditional Document-level GN	33.5	75.9	46.5

## Discussion

The above experimental results indicate that the proposed methods, including the refinement process and the intra/cross-section collective approaches, can significantly increase the number of normalized gene mentions. Nevertheless, after a thorough analysis of the results, it was found that these methods also increased the false positive rate, and this increase is more evident at the instance level rather than the document level.

**Figure 7:**
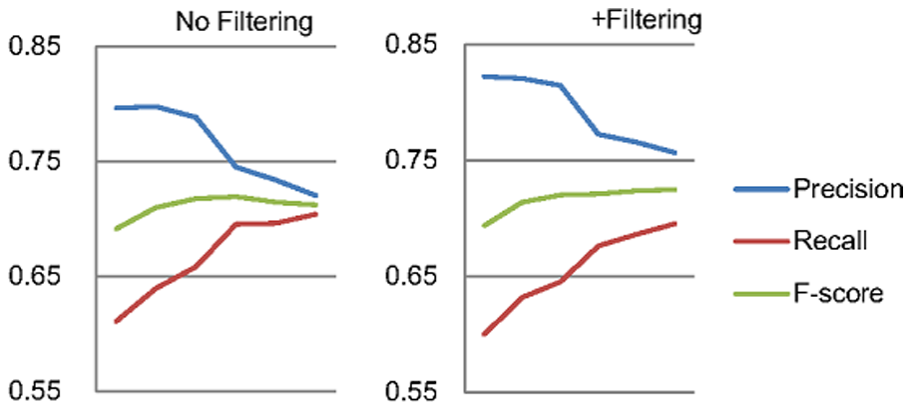
The effort of adding more individual disambiguation formulae.

Figure 7 delineates this issue, in which the horizontal axis is the numbers of added individual formula sets, and the vertical axis is the achieved scores. As shown in the left part of the figure, adding more individual formulae in our collective GN model can greatly boost the recall, but significantly reduce the precision if we exclude Formula 5 and its related formulae. This is caused by the normalization of false positive mentions recognized by the entity recognition and refinement steps. For example, consider the sentence “It is located on the short arm of chromosome 1 in the region **1p34** and **p35**”. The two surface names “1p34” and “p35” are very likely to be recognized as gene mentions because of the orthographical features. The normalization of these mentions is highly probable when more disambiguation formulae are added. Under the setting of the instance-level GN evaluation and our formulation, this issue becomes even more critical since such errors will be propagated to other false positive cases through the inter- and cross-section formulae, and will then be penalized by the instance-level evaluation scheme. As for the majority of entity normalization works in the general domain (*e.g.* Cucerzan, 2007; Kulkarni et al., 2009; Rada Mihalcea & Csomai, 2007), the same surface name described in an article is assumed to always refer to the same instance. Nonetheless, based on our analysis on the IGN corpus, 14.9% of the articles contain different gene mentions that possess the same surface name, which lead to an average of 2.93 assigned Entrez Gene ID per name. Therefore, a filtering mechanism plays an important role in our approach. The right part of Figure 7 shows the proposed filtering constraint (Formula 5) can somewhat ameliorate this problem and result in an improved F-score.

Currently, the effect of our proposed method is only substantiated on the human gene. We believe that the issues brought up will be even more challenging when considering cross-species GN, and investigating the effect of our intra- and cross-section collective GN formulation for this task will be a very intriguing topic. We can foresee that more formulae will be required, not only for filtering but also for other issues, such as precisely normalizing one gene mention to mulitple IDs in a narration like “The site is conserved in the human, rat, and mouse p53 promoters”. The emerging larger Markov network models can make the inference a computationally challenging problem, and advanced inference algorithms are required to prune the unnecessary nodes in order to improve the inference efficiency.

## Conclusions

In this work, we compile the first instance-based GN corpus and present a collective classification approach to deal with the instance-based GN challenges at the abstract and full text levels. The released corpus contains annotations for gene mentions, their linked IDs along with dependency information, such as co-reference chains and full name/abbreviation pairs, which have the potential to significantly advance instance level GN research. We propose Markov logic formulae to model dependencies among gene mentions and mentions across different sections. These formulae exploit the linguistic phenomena and the inherent characteristics of paper sections annotated in the IGN corpus, and the experimental results demonstrate the advantages. In addition, a refinement process is proposed, which can significantly improve the entity recognition and normalization performance. We believe that these results may benefit current and new GN researchers. The ultimate goal of life science researches nowadays is to reveal mechanisms underlying biological phenomenon, with a hope of benefitting human beings in different ways. Throughout our collaborations with life scientists, basically all those that utilizes the GN technique requires us to link the gene names to its human ID, which is why we have decided to emphasize our results on human genes in the current study. We expect our approach to efficiently assist life scientists in identifying individual gene mentions, with the hope of acquiring a comprehensive overview of biological pathways and mechanisms enclosed within biomedical literatures. In the future, we will improve the IGN corpus by filling-in the ID information for non-human genes, and extend the proposed collective approach to conduct cross species GN. We will also integrate the developed GN system into our online web application, PubMed-EX [35], and view its potential in processing the large-scale data.

## Supporting Information

Material S1 **Gene Normalization Markov Logic Formulae.**
(DOCX)
