# Correction: Collective Instance-Level Gene Normalization on the IGN Corpus

**DOI:** 10.1371/annotation/3c124386-b718-40d9-bc05-495190f9a184

**Published:** 2014-01-02

**Authors:** Hong-Jie Dai, Johnny Chi-Yang Wu, Richard Tzong-Han Tsai

A funding organization and grant were incorrectly omitted from the Funding Statement. The Funding Statement should read: "This research was supported by the National Science Council of Taiwan under Grant NSC-102-2218-E-038-001 and the research grant TMU101-AE1-B55 of Taipei Medical University. The funders had no role in study design, data collection and analysis, decision to publish, or preparation of the manuscript." 

