# Supplementary material for: Collective Instance-Level Gene Normalization on the IGN Corpus
Source: PLoS One. 2013 Nov 25;8(11):e79517. doi: 10.1371/journal.pone.0079517 (PMC3839972; doi:10.1371/journal.pone.0079517)
Supplement: Material S1 — Gene Normalization Markov Logic Formulae. (DOCX) [file pone.0079517.s001.docx]

## Gene Normalization Markov Logic Formulae

### Gene Normalization Formulae

#### Individual Formulae

$$Candidate(x, id)\wedge ChromosomeInfo\left( x, id, +sd \right)\Longrightarrow NormalizeTo(x, id)$$

$$HasWord\left( w \right)\wedge PPIKeyword\left( w \right)\wedge Candidate\left( x, id \right)\wedge\exists!{id}_{i}.MostPPIPartners\left( {id}_{i} \right)\wedge{id}_{i}=id\Longrightarrow NormalizeTo\left( x, id \right)$$

$$Candidate\left( x, id \right)\wedge GOTermsSmithWatermanScore\left( x, id, score \right)\wedge score>30\Longrightarrow NormalizeTo\left( x, id \right)$$

$$Candidate\left( x, id \right)\wedge\exists!{id}_{i}.MostGOTerms\left( {id}_{i} \right)\wedge{id}_{i}=id\Longrightarrow NormalizeTo\left( x, id \right)$$

$$Candidate\left( x, id \right)\wedge\exists!{id}_{i}.MostTissueTerms\left( {id}_{i} \right)\wedge{id}_{i}=id\Longrightarrow NormalizeTo\left( x, id \right)$$

$$Candidate\left( x, id \right)\wedge\exists!{id}_{i}.MostLexiconMatchingCount\left( {id}_{i} \right)\wedge{id}_{i}=id\Longrightarrow NormalizeTo\left( x, id \right)$$

$$\exists!id.Candidate\left( x, id \right)\Longrightarrow NormalizeTo\left( x, id \right)$$

$$Candidate\left( x, id \right)\wedge\exists!{id}_{i}.MostLexiconMatchingCount\left( {id}_{i} \right)\wedge{id}_{i}=id\Longrightarrow NormalizeTo(x, id)$$

$$\exists!u,id.Candidate\left( x, id \right)\wedge PrecedingWord\left( x+1, u \right)\wedge u=\text{(}\wedge UnigramBetween\left( x,x+1, u \right)\wedge FollowingWord\left( x+1, "\text{)"} \right)\Longrightarrow NormalizeTo(x+1, id)$$

$$\exists!u,id.Candidate(x+1, id)\wedge PrecedingWord\left( x+1, u \right)\wedge u="("\wedge UnigramBetween\left( x,x+1, u \right)\wedge FollowingWord\left( x+1, "\text{)"} \right)\Longrightarrow NormalizeTo(x, id)$$

#### Collective Formulae

##### Intra-section Collectives

$$HasWord\left( w \right)\wedge PPIKeyword\left( w \right)\wedge NormalizeTo(x, {id}_{i})\wedge Candidate(y,{id}_{j})\wedge PPIPartner({id}_{i}, {id}_{j})\Longrightarrow NormalizeTo\left( j, {id}_{j} \right)$$

$$Precede\left( x, y \right)\wedge NormalizeTo(x,id)\wedge Candidate\left( y, id, s \right)\Longrightarrow NormalizeTo(y,id)$$

$$\exists!u.u="("\wedge PrecedingWord\left( x+1, u \right)\wedge UnigramBetween\left( x,x+1, u \right)\wedge FollowingWord\left( x+1, "\text{)"} \right)\wedge NormalizeTo\left( x, id \right)\Longrightarrow NormalizeTo(x+1, id)$$

$$\exists!u.u="("\wedge PrecedingWord\left( x+1, u \right)\wedge UnigramBetween\left( x,x+1, u \right)\wedge FollowingWord\left( x+1, "\text{)"} \right)\wedge NormalizeTo\left( x+1, id \right)\Longrightarrow NormalizeTo(x, id)$$

$$Coreference\left( x, y \right)\wedge NormalizeTo\left( x, {id}_{i} \right)\wedge\neg\exists{id}_{j}.NormalizeTo\left( y, {id}_{j} \right)\Longrightarrow NormalizeTo\left( y, {id}_{i} \right)$$

##### Cross-section Collectives

$$InfoRichSection\left( s_{i} \right)\wedge NormalizeTo(x,id)\wedge Candidate\left( y, id, s_{j} \right)\wedge s_{i}\neq s_{j}\Longrightarrow NormalizeTo(y,id)$$

$$Coreference\left( x, y \right)\wedge NormalizeTo\left( x, {id}_{i} \right)\wedge\neg\exists{id}_{j}.NormalizeTo\left( y, {id}_{j} \right)\wedge Name\left( x, \_,\boldsymbol{s}_{\boldsymbol{i}} \right)\wedge InfoRichSection\left( s_{i} \right)\wedge s_{i}\neq s_{j}\wedge Name\left( y, \_,s_{j} \right)\Longrightarrow NormalizeTo\left( y, {id}_{i} \right)$$

$$HasWord\left( w \right)\wedge PPIKeyword\left( w \right)\wedge NormalizeTo(x, {id}_{i})\wedge Name(x,\_, s_{i})\wedge InfoRichSection\left( s_{i} \right)\wedge s_{i}\neq s_{j}\wedge Candidate\left( y, {id}_{j}, s_{j} \right)\wedge PPIPartner({id}_{i}, {id}_{j})\Longrightarrow NormalizeTo\left( y, {id}_{j} \right)$$

#### Gene Normalization Constraints

$$NormalizeTo(x, {id}_{i})\wedge{id}_{i}\neq{id}_{j}\Longrightarrow\neg NormalizeTo(x, {id}_{j})$$

$$HumanGene\left( x \right)\vee\neg NormalizeTo(x, id)$$

### Co-reference Resolution Formulae

$$Name\left( x, n, \_ \right)\wedge Name(y, n, \_)\Longrightarrow Coreference(x, y)$$

$$SynonymOf\left( x, y, \_ \right)\Longrightarrow Coreference(x, y)$$

$$PatternNormalizedName\left( x, n, \_ \right)\wedge PatternNormalizedName(y, n, \_)\Longrightarrow Coreference(x, y)$$

$$AliasOf\left( x, y \right)\wedge Name\left( x, n, \_ \right)\wedge Name\left( y, n,\_ \right)\Longrightarrow Coreference(x, y)$$

$$Distance(x, y, +d))\Longrightarrow Coreference(x, y)$$

$$AppositionTo(x, y)\Longrightarrow Coreference(x, y)$$

#### Co-reference Constraint

$$Coreference(x, y)\Longrightarrow HumanGene\left( x \right)\wedge HumanGene\left( y \right)$$

$$\forall x.Coreference(x, x)$$

$$\forall x, y.Coreference(x, y)\Longrightarrow Coreference(y, x)$$

$$\forall x, y,z.Coreference(x, y)\bigwedge Coreference(y, z)\Longrightarrow Coreference(x, z)$$

### Human Gene Classification Formulae

$$Name(x, +n, s)\Longrightarrow HumanGene\left( x \right)$$

$$FirstWord\left( x, +w \right)\wedge SpeciesTerm\left( +w \right)\Longrightarrow HumanGene\left( x \right)$$

$$PrecedingWord\left( x, +w \right)\wedge SpeciesTerm\left( +w \right)\Longrightarrow HumanGene\left( x \right)$$

$$LastWord\left( x, +w \right)\wedge Blacklisted\left( +w \right)\Longrightarrow\neg HumanGene\left( x \right)$$

$$FollowingWord\left( x, +w \right)\wedge Blacklisted\left( +w \right)\Longrightarrow\neg HumanGene\left( x \right)$$

$$Name(x, +n, s)\wedge Blacklisted(+n)\Longrightarrow\neg HumanGene\left( x \right)$$

$$Name(x, n, s)\wedge AllUpperCases(n)\wedge Blacklisted(n)\wedge PrecedingWord\left( x, "\text{("}, 1 \right)\wedge Precede(y, x)\wedge\exists!u.UnigramBetween\left( x,y, u \right)\wedge u="("\Longrightarrow\neg HumanGene(y, id)$$

$$Name(x, n, s)\wedge AllUpperCases(n)\wedge Blacklisted(n)\wedge FollowingWord\left( x, "\text{(}\text{"}, 1 \right)\wedge Precede(x, y)\wedge\exists!u.UnigramBetween\left( x,y, u \right)\wedge u="("\Longrightarrow\neg HumanGene(y, id)$$

$$Name(x, n, s)\wedge Blacklisted(n)\wedge FollowingWord\left( x, "(", 1 \right)\wedge\exists!u.UnigramBetween\left( x,y, u \right)\wedge u="("\Longrightarrow\neg HumanGene(y, id)$$

$$Name(x, n, s)\wedge Blacklisted(n)\wedge PrecedingWord\left( x, "(", 1 \right)\wedge\exists!u.UnigramBetween\left( x,y, u \right)\wedge u="("\Longrightarrow\neg HumanGene(y, id)$$

$$MoreSpecificMentions\left( x \right)\Longrightarrow\neg HumanGene\left( x \right)$$

$$\neg\exists id.Candidate\left( x, id \right)\Longrightarrow\neg HumanGene\left( x \right)$$

$$\exists!id.Candidate\left( x, id \right)\Longrightarrow HumanGene\left( x, id \right)$$

$$Candidate\left( x, id \right)\Longrightarrow HumanGene\left( x, id \right)$$
